# Setting Up an Out of Hours Supervision Group at St Charles Mental Health Unit

**DOI:** 10.1192/bjo.2022.158

**Published:** 2022-06-20

**Authors:** Win Thet, Hamish Naismith, Mehtab Rahman

**Affiliations:** Central and North West London NHS Foundation Trust, London, United Kingdom

## Abstract

**Aims:**

Psychiatric on-calls are often regarded as the most challenging aspect of core psychiatric training. This audit aimed to gain trainee feedback about on-calls at one of London's busiest mental health units, whether they were receiving adequate supervision for emergency and out of hours work and to design an intervention to improve on-call supervision experience for core and higher trainees.

**Methods:**

A qualitative survey to assess the out of hours clinical experiences of trainees was conducted. The survey explored the following domains: trainees’ confidence in dealing with emergencies out of hours, quality of supervision and individual learning opportunities.

**Results:**

Results indicated low to moderate confidence levels among trainees in performing out of hours’ clinical tasks. The majority were of the opinion that further supervision for on-calls would be beneficial. 59% of trainees stated they struggled to complete work place based assessments (WPBAs) on out of hours cases. In view of the findings, a quality improvement framework was used to introduce a supervision group that gave trainees the opportunity to learn from their out of hours complex cases with a Consultant Psychiatrist as a chair. Following the implementation of the group, a qualitative survey revealed improved confidence, morale and training satisfaction among trainees. The results of the survey and feedback from trainees will be shared in details in the poster. This group has been running successfully for the last one year.

**Conclusion:**

The introduction of an out of hours supervision group in busy mental health units can lead to an improvement in confidence and enable professional and educational development for trainees, which will also help improve overall morale as evidenced by this audit. Additional supervision and developing confidence of junior doctors in dealing with out of hours’ complex cases has enabled trainees to feel more supported and has led to increased training satisfaction at St Charles Hospital, London.

